# Ameliorative effects of *Scutellaria Pinnatifida* subsp. *pichleri* (Stapf) Rech.f. Extract in streptozotocin-induced diabetic rats: chemical composition, biochemical and histopathological evaluation

**DOI:** 10.1186/s12906-023-04252-w

**Published:** 2023-11-14

**Authors:** Mehmet Salih Bakaç, Abdulahad Dogan, Mustafa Abdullah Yılmaz, Fikret Altındag, Fatih Donmez, Abdulhamit Battal

**Affiliations:** 1 Department of Basic Sciences Pharmacy, Institute of Health Sciences, Van Yuzuncu Yil University, Van, Turkey; 2 Department of Biochemistry, Faculty of Pharmacy, Van Yuzuncu Yil University, Van, 650080 Turkey; 3 Department of Pharmaceutical Chemistry, Faculty of Pharmacy, Dicle University, Diyarbakır, Turkey; 4 Department of Medical Histology and Embryology, Faculty of Medicine, Van Yuzuncu Yil University, Van, Turkey; 5 Department of Pharmaceutical Biotechnology, Faculty of Pharmacy, Van Yuzuncu Yil University, Van, Turkey

**Keywords:** *Scutellaria Pinnatifida* subsp. *pichleri*, Apigenin, Antioxidant, Phenolic content, Diabetes

## Abstract

**Backgrounds:**

*Scutellaria Pinnatifida* subsp. *pichleri* (Stapf) Rech.f. (SP) is used in folk medicine for the treatment of diabetes. The aim of the study was to determine the phenolic profile of SP extract (SPE) by LC-MS/MS and to investigate the antidiabetic, hepatoprotective and nephroprotective effects of SPE in streptozotosin (STZ)-induced diabetic rat model.

**Methods:**

Forty-two rats were randomly divided into six groups (n = 7): Control (nondiabetic), diabetes mellitus (DM), DM + SP-100 (diabetic rats treated with SPE, 100 mg/kg/day), DM + SP-200 (diabetic rats treated with SPE, 200 mg/kg/day), DM + SP-400 (diabetic rats treated with SPE, 400 mg/kg/day) and DM + Gly-3 (diabetic rats treated with glibenclamide, 3 mg/kg/day). Live body weight, fasting blood glucose (FBG) level, antidiabetic, serum biochemical and lipid profile parameters, antioxidant defense system, malondyaldehyde (MDA) and histopathological examinations in liver, kidney and pancreas were evaluated.

**Results:**

Apigenin, luteolin, quinic acid, cosmosiin and epigallocatechin were determined to be the major phenolic compounds in the SPE. Administration of the highest dose of SP extract (400 mg/kg) resulted in a significant reduction in FBG levels and glycosylated hemoglobin levels in STZ-induced diabetic rats, indicating an antihyperglycemic effect. SPE (200 and 400 mg/kg) and glibenclamide significantly improved MDA in liver and kidney tissues. In addition, SPE contributed to the struggle against STZ-induced oxidative stress by stimulating antioxidant defense systems. STZ induction negatively affected liver, kidney and pancreas tissues according to histopathological findings. Treatment with 400 mg/kg and glibenclamide attenuated these negative effects.

**Conclusions:**

In conclusion, the extract of the aerial part of *Scutellaria pinnatifida* subsp. *pichleri* has hepatoprotective, nephroprotective and insulin secretion stimulating effects against STZ-induced diabetes and its complications due to its antidiabetic and antioxidant phytochemicals such as apigenin, luteolin, quinic acid, cosmosiin and epigallocatechin.

## Highlights

*Scutellaria pinnatifida* subsp. *pichleri* ethanolic extract (SPE) has apigenin, luteolin, quinic acid, cosmosiin and epigallocatechin as the major phenolic compounds.

400 mg/kg SPE treatment regulated glucose level and glycosylated hemoglobin.

SPE contributed to antioxidant defense system.

SPE improved to islet volume density of Langerhans.

## Introduction

Diabetes, also known as diabetes mellitus (DM), is an endocrinological condition defined by a fasting blood glucose level greater than 126 mg/dL caused by inadequate or insufficient insulin release from the ß-cells in the Langerhans of the pancreas [1]. The number of patients with diabetes is increasing every year. In the 2000s, 171 million people had diabetes; in 2019, 425 million people will have diabetes and it is expected that 643 million people will be affected in 2030 and 783 million people in 2045 if the necessary precautions are not taken in time [2]. While the global health expenditure on diabetes was $760 billion in 2019, this amount is expected to increase to $825 billion in 2030 and $845 billion in 2045 [2]. Diabetes is classified into four types: Type 1, Type 2, gestational diabetes, and diabetes triggered by other factors. However, the etiological classification of diabetes is mainly divided into two main groups, type 1 and type 2, with type 2 diabetes being the vast majority (90%) of the total prevalence of DM [3]. It is known that the development of DM is influenced by a number of factors, including diet, physical inactivity, environmental toxins, stress, insomnia, and genetic predisposition. DM can cause both microvascular (retinopathy, nephropathy, and neuropathy) and macrovascular (ischemic heart disease, stroke, and peripheral vascular disorders) multisystem consequences [4].

Increasing reactive oxygen species (ROS) and reactive nitrogen species (RNS) in the cell cause oxidative stress (OS). The OS plays an important role in the development of diabetes and diabetic complications. Hyperglycemia causes the production of ROS [5]. Increased ROS causes cellular damage by attacking carbohydrates, proteins, lipids and nucleic acids [6]. Antioxidants are used to minimize or eliminate the damage caused by ROS and RNS.

According to the World Health Organization (WHO), there are approximately 21,000 medicinal plants in the world [7]. Based on diabetic animal models and in vitro biological activity results, it has been reported that 800 plant species have antidiabetic potential [8]. Plant extracts rich in polyphenols, alkaloids, glycosides, flavonoids and terpenoids have healing effects on diabetes and its complications [9]. Plants or their bioactive compounds can have antidiabetic effects in several ways. These include enhancing insulin synthesis, insulin sensitivity, and secretion from pancreatic β-cells, as well as stimulating pancreatic regeneration or rejuvenation, reducing gastrointestinal glucose uptake, insulin mimicking properties, and altering the activity of various associated enzymes [10].

The Lamiaceae family includes 360–469 species of the genus *Scutellaria*, which is widely distributed in Europe, North America, East Asia, and South America [11]. *Scutellaria* species are rich in secondary plant compounds such as flavonoids, phenylethanoid glycosides, iridoid glycosides, diterpenes and triterpenoids, alkaloids, phytosterols and polysaccharides [12]. In addition, some *Scutellaria* species have been found to have potential benefits in the treatment of metabolic syndromes and related diseases such as obesity, hyperlipidemia, atherosclerosis, diabetes, and their complications [13–15].

The genus *Scutellaria*, commonly known as skullcaps, is widely used in traditional medicine in various countries such as China, North America, Korea and Europe [16]. The SP plant, which has high phenolic and flavonoid content, exhibits antibacterial, antioxidant, antiviral, hepatoprotective, anti-inflammatory, neuroprotective, anticancer, antidiabetic effects and scavenges free radicals [12, 17–22].

The aerial parts of *Scutellaria orientalis* subsp. *pichleri* (Stapf) J.R.Edm. synonym of *Scutellaria pinnatifida* subsp. *pichleri* (Stapf) Rech.f. (The plant name corresponds to the latest revision in “World Flora Online”. http://www.worldfloraonline.org/taxon/wfo-0000308297) are used in folk medicine to treat diabetes in Van province, in Turkey [23]. *Scutellaria orientalis* L. inhibits α-amylase and α-glucosidase, which are important parameters for determining antidiabetic potential in vitro [24]. In addition, wogonin, baicalein, chrysin and apigenin contained in the aerial part of the *Scutellaria orientalis* L. are potent antidiabetic phenolic compounds [25–28]. Moreover, the methanolic and dichloromethane extracts of the aerial parts of SP contain luteolin-7-o-glucoside, apigenin-7-o-glucoside, phlomisethanoside, syringalide A, verbascoside and oleic acid [29]. The antidiabetic properties of luteolin, apigenin, verbascoside and oleic acid have been reported [28, 30–32]. There are few in vitro studies on *Scutellaria pinnatifida* subsp. *pichleri*, but there is no in vivo study on diabetes. This is the first study to investigate the ameliorative effect of the plant *Scutellaria pinnatifida* subsp. *pichleri* in the STZ-induced rat model. The aim of this study was to identify the phenolic acid profile of ethanolic extract of *S. pinnatifida* plant by LC-MS/MS and to comprehensively investigate its antidiabetic, hepatoprotective, and nephroprotective effects in a STZ-induced diabetic rat model in terms of biochemical, antioxidant, and histological findings.

## Materials and methods

### Reagents

All chemicals and reagents used in this study were obtained from Sigma Chemical Co. (St. Louis, MO, USA). ELISA kits for glutathione peroxidase (catalogue number: EK720976) and superoxide dismutase (catalogue number: EK720188) were purchased from AFG Bioscience LLC (Northbrook, USA).

### Plant and ethanolic lyophilized extract preparation

The aerial parts of the plant *Scutellaria pinnatifida* subsp. *pichleri* (Stapf) Rech.f. were used for the study. The plant was collected by Assoc. Dr. Abdulahad DOĞAN in Van Gürpınar province, Turkey, May 2022. Assoc. Prof. Dr. Hüseyin EROĞLU of Van Yuzuncu Yıl University, Department of Biology, Department of Botany identified the plant. One voucher specimen (Herbarium number: 165,215) has been deposited in the Herbarium (VANF) of Yüzüncü Yıl University. Lyophilized ethanolic extracts of the aboveground plant were prepared according to Dogan et al. [33]. Briefly, dried above-ground plant material was cut into small pieces, extracted into 80% ethanol and filtrated. Ethanol was evaporated using a rotavapor and water was removed from the frozen extract using the freeze-drying process to obtain a freeze-dried ethanolic extract.

### Total phenolic and flavonoid contents in the SP extract

The total phenolic content of *S. pinnatifida* extract (SPE) was determined by mg/g gallic acid equivalent (GAE) according to the gallic acid standard curve (y = 0.5752x − 0.0059, R2 = 0.9951) using Folin-Ciocalteu phenol reagent and sodium carbonate solution [34]. The total flavonoid content of SPE was determined by mg/g quercetin equivalent (QE) according to the quercetin standard curve (y = 3.2461x + 0.0138, R2 = 0.9941) [35].

### Liquid chromatography-mass spectrometry/mass spectrometry (LC-MS/MS) analysis of the SP extract

The phenolic compounds present in the SPE were identified and quantified by LC-MS/MS analysis, which was previously validated for 53 compounds on an ultra-high performance liquid chromatography instrument [36]. Ferulic acid D1 (20 mg/L), rutin D2 (1 mg/L) and quercetin D3 (5 mg/L) were used as internal standards (IS).

### Animals

For the experimental study, a total of 48 male *Wistar albino* rats aged 2–3 months, with body weights between 150 and 250 g were obtained from the Experimental Animals Unit of Van Yüzüncü Yıl University, Van, Turkey. Six rats were used for toxicity test and the remaining forty-two were used for experimental study. Prior to the experiments, rats were acclimatized to conditions in the experimental laboratory for 7 days and fed *ad libitum* in standard plastic material cages with a stainless steel kept at room temperature (photoperiod: 12:12 h light/dark period). For ethical issues, the directive ARRIVE guidelines (Directive 2010/63/EU) [37] was followed in this study. In addition, the animals were handled in accordance with the requirements of the Van Yüzüncü Yıl University Experimental Animal Unit’s Institutional Committee of Care and Use of Laboratory Animals, which approved the experiments under the decision of 01/12/2022 and number 2022/12–18.

### Toxicity test and determination of SP extract doses

Six male *Wistar albino* rats were used for the toxicity test. The toxicity test was performed according to Organization for Economic Cooperation and Development (OECD) protocol 432. The rats were treated with 50, 300 and 2000 mg/kg SPE by the oral route. No morbidity or mortality was observed in the rats during the toxicity test. 100, 200 and 400 mg/kg SPE were set as experimental doses.

### Induction of diabetes and experimental design

Rats were treated with a single intraperitoneal (i.p.) dose of 50 mg/kg streptozotocin (STZ) dissolved in 0.1 M cold chilled citrate buffer (pH:4.5) to induce a diabetic rat model [38]. Tail blood glucose levels were measured 72 h after administration using a glucometer to determine whether the rats were diabetic or not. The rats were classified as diabetic if their blood glucose level was higher than 200 mg/dL.

Grouping was based on the live weight of rats. The groups had normal distribution according to the Kolmogorov-Smirnov test (P > 0.05). Forty-two male *Wistar albino* rats were divided into six groups (n = 7 per group), including a total of 35 that had successfully induced diabetes mellitus, while 7 served as healthy control as follows:

(I) Control group (n = 7): Rats were fed standard feed and water *ad libitum* throughout the experiment. In addition, a once daily dose (1 ml/kg) of physiological water was administered by gavage.
(II) Diabetes mellitus (DM) group (n = 7): Diabetic rats that were STZ-induced were fed standard feed and water *ad libitum* throughout the experiment. In addition, a once daily dose (1 ml/kg) of physiological water was administered by gavage.
(III) DM + SP-100 mg/kg extract group (n = 7): Diabetic rats that were STZ-induced were fed standard feed and water *ad libitum* throughout the experiment. In addition, diabetic rats were administered an extract of *S. pinnatifida* (100 mg/kg/day extract) by gavage according to their body weight (bw) during the experiment.
(IV) DM + SP-200 mg/kg extract group (n = 7): Diabetic rats that were STZ-induced were fed standard feed and water *ad libitum* throughout the experiment. In addition, diabetic rats were administered an extract of *S. pinnatifida* (200 mg/kg/day extract) by gavage according to their bw during the experiment.
(V) DM + SP-400 mg/kg extract group (n = 7): Diabetic rats that were STZ-induced were fed standard feed and water *ad libitum* throughout the experiment. In addition, diabetic rats were administered an extract of *S. pinnatifida* (400 mg/kg/day extract) by gavage according to their bw during the experiment.
(VI) DM + Gly-3 mg/kg group (n = 7): Diabetic rats that were STZ-induced were fed standard feed and water *ad libitum* throughout the experiment. In addition, glibenclamide (3 mg/kg/day), an oral antidiabetic drug, was administered by gavage to diabetic rats, according to their bw during experiment.

Rats were weighed to determine live body weight (LBW) and fasting blood glucose (FBG) level was measured at the tail for each week.

### Tissue homogenization

This study was conducted for 21 days. Rats were anesthetized i.p. with a combination of ketamine HCl (50 mg/kg) and xylazine HCl (10 mg/kg). The depth of anesthesia was determined by toe squeezing. Rats were euthanized by the cardiac puncture method (collection of blood from the heart) with an injector under deep anesthesia [38]. Blood was transferred into hemogram and biochemistry tubes. Then, the tubes were centrifuged at 3000 RPM at room temperature for 10 min and serum was collected. Liver, kidney and pancreas tissues were removed and stored in the ultra-freezer until analysis.

A buffer with a pH of 7.4 and ingredients including 50 mM KH_2_PO_4_ and 1 mmol/L EDTA was used for the extraction [33]. Then, 5 mL of the cold buffer was added after 500 mg of tissue was weighed. Tissues were minced with a glass rod before being thoroughly homogenized for three minutes in an ultrasonic homogenizer (SONOPULS HD 2200, Bandelin, Berlin, Germany). The extract was immediately centrifuged in a refrigerated centrifuge (+ 4 °C) for 30 min at 9500 RPM. Lipid peroxidation and antioxidant enzyme assays were then performed on the supernatants from the liver, kidney, and pancreas.

### Serum biochemical parameter analysis

The parameters aspartate aminotransferase (AST), alanine aminotransferase (ALT), lactate dehydrogenase (LDH), creatinine, urea, glucose, and lipid profile [total cholesterol (TC) and high-density lipoptotein_cholesterol (HDL_c)] parameters were determined with the Cobas 6000 Autoanalyzer instrument (Roche Diagnostics GmBH, Mannheim, Germany). The percentage of glycosylated hemoglobin (HbA1c %) in total blood was measured using Tina-quant hemoglobin A1c Gen.3 kits from COBAS INTEGRA/cobas c systems, (Roche Diagnostics, Indianapolis, Germany).

### Protein, malondialdehyde, reduced glutathione, and antioxidant enzyme analyzes

The total protein content of the tissue was determined using the Bradford technique [39]. The concentration of malondialdehyde (MDA) in the tissues was determined using the thiobarbituric acid reactivity method developed by Buege and Aust [40]. The concentration of reduced glutathione (GSH) in tissues was described according to Beutler et al. [41]. Glutathione S-transferase (GST) activity was measured by monitoring the conjugation of glutathione with 1-chloro-2,4-dinitrobenzene at 340 nm, as described by Mannervik and Guthenberg [42]. Glutathione reductase activity (GR) was measured according to Carlberg and Mannervik [43]. Catalase (CAT) activity was determined according to the method described by Aebi [44]. The activities of glutathione peroxidase (GPx) and superoxide dismutase (SOD) enzymes were measured according to the protocol of ELISA kits from AFG Bioscience LLC (Northbrook, USA).

### Histopathological examinations

Liver, kidney and pancreas tissues from sacrificed rats were fixed in 10% neutral-buffered formalin. The fixed tissues were dehydrated in alcohol and purified in xylene using an automated tissue tracking device (LEICA ASP300S). Liver, kidney and pancreas tissues were embedded in the paraffin. Sections of 5 μm thickness were taken from the paraffin blocks using microtome. Sections were stained with Hematoxylin-Eosin and examined under a light microscope (Olympus BX53, Japan). Histopathological findings were quantified according to Khanal and Patil [45].

### The volume density of the pancreatic islets

The Cavalieri method, a stereological analysis method, was used to calculate the volume density of the pancreatic islets. Therefore, the total number of points hitting the pancreas and the total number of points hitting the pancreatic islets were counted. The volume was calculated by multiplying the total number of points by the area covered by a point and the section thickness. Thus, the volume of the entire pancreas and pancreatic islets was calculated. The following formula was used to calculate the volume; V = ΣP x a/p x t, where ‘‘V’’ is the volume of the structure, ‘‘ΣP′′ is the total number of points hitting the structure, ‘‘a/p’’ is the area covered by one point, and ‘‘t’’ is the cross-section thickness. The following formula was used for the volume density of the pancreatic islets; V_v(structure/ref)_ = V_(structure)_/V_(total)_ [46, 47]. Where ‘‘V_(structure)_’’ is the volume of the related structure, ‘‘V_(total)_’’ is the total volume of the tissue.

### Statistical analysis

Data obtained from the study were presented as mean ± standard error of mean (SEM). GraphPad Prism 8 was used to analyze the data. For comparison of body weight and blood glucose level parameters, a paired t-test was used as a statistical analysis. Control group was compared with the DM group and DM group was compared with all treatment groups. Statistical differences were determined using one-way analysis of variance (ANOVA) and Tukey’s multiple comparison post hoc test. The significant value accepted was *P* < 0.05 for all tests.

## Results

### Total phenolic and flavonoid contents of the SP extract

The yield of *S. pinnatifida* extract (SPE) was found to be 18.25%. Total phenolic and total flavonoid contents were determined to be 61.34 ± 3.29 mg GAE/g and 48.60 ± 2.68 mg RE/g, respectively.

### Identification and quantification of phenolic acids in SP extract by LC–MS/MS

**Fig. 1:**
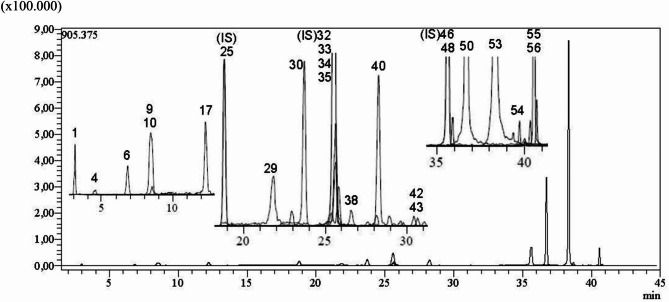
Quantitative screening chromatogram of phenolic acids in the ethanol extract of the SP by LC–MS/MS. 1: Quinic acid, 4: Gallic acid, 6: Protocatechuic acid, 9: Chlorogenic acid, 10: Protocatechuic aldehyde, 17: Caffeic acid, 25: Ferulic acid-D3-IS^*h*^, 29: Salicylic acid, 30: Cynaroside, 32: Rutin-D3-IS^*h*^, 33: Rutin, 34: Isoquercitrin, 35: Hesperidin, 38: Rosmarinic acid, 40: Cosmosiin, 42: Astragalin, 43: Nicotiflorin, 47: Quercetin-D3-IS^*h*^, 48: Naringenin, 50: Luteolin, 53: Apigenin, 54: Amentoflavone, 55: Chrysin and 56: Acacetin

The phenolic compounds were identified based on retention time as follows: Quinic acid, gallic acid, protocatechuic acid, chlorogenic acid, protocatechuic aldehyde, caffeic acid, salicylic acid, cynaroside, rutin, isoquercitrin, hesperidin, rosmarinic acid, cosmosiin, astragalin, nicotiflorin, naringenin, luteolin, apigenin, amentoflavone, chrysin and acacetin (Fig. 1). Phenolic acids in SPE were quantified as µg analyte/g extract (Table 1). The major compounds in SPE are apigenin, luteolin, quinic acid, cosmosiin and epigallocatechin.

**Table 1:** Quantitative screening of phenolic acids in ethanol extract of the SP by LC–MS/MS

No	Compounds	RT	Amount (µg analyte/ g extract)	No	Compounds	RT	Amount (µg analyte/ g extract)
1	Quinic acid	3.0	0.701	29	Salicylic acid	21.8	0.045
2	Fumaric aid	3.9	ND	30	Cynaroside	23.7	0.617
3	Aconitic acid	4.0	ND	31	Miquelianin	24.1	ND
4	Gallic acid	4.4	0.026	32	Rutin-D3-IS^*h*^	25.5	NA
5	Epigallocatechin	6.7	ND	33	Rutin	25.6	0.625
6	Protocatechuic acid	6.8	0.155	34	Isoquercitrin	25.6	0.249
7	Catechin	7.4	ND	35	Hesperidin	25.8	0.381
8	Gentisic acid	8.3	ND	36	*o*-Coumaric acid	26.1	ND
9	Chlorogenic acid	8.4	0.283	37	Genistin	26.3	ND
10	Protocatechuic aldehyde	8.5	0.021	38	Rosmarinic acid	26.6	0.087
11	Tannic acid	9.2	ND	39	Ellagic acid	27.6	ND
12	Epigallocatechin gallate	9.4	ND	40	Cosmosiin	28.2	0.656
13	1,5-dicaffeoylquinic acid	9.8	ND	41	Quercitrin	29.8	ND
14	4-OH Benzoic acid	10.5	ND	42	Astragalin	30.4	0.108
15	Epicatechin	11.6	ND	43	Nicotiflorin	30.6	0.051
16	Vanilic acid	11.8	ND	44	Fisetin	30.6	ND
17	Caffeic acid	12.1	0.077	45	Daidzein	34.0	ND
18	Syringic acid	12.6	ND	46	Quercetin-D3-IS^*h*^	35.6	NA
19	Vanillin	13.9	ND	47	Quercetin	35.7	ND
20	Syringic aldehyde	14.6	ND	48	Naringenin	35.9	0.040
21	Daidzin	15.2	ND	49	Hesperetin	36.7	ND
22	Epicatechin gallate	15.5	ND	50	Luteolin	36.7	0.932
23	Piceid	17.2	ND	51	Genistein	36.9	ND
24	*p*-Coumaric acid	17.8	ND	52	Kaempferol	37.9	ND
25	Ferulic acid-D3-IS^*h*^	18.8	NA	53	Apigenin	38.2	1.653
26	Ferulic acid	18.8	ND	54	Amentoflavone	39.7	0.002
27	Sinapic acid	18.9	ND	55	Chrysin	40.5	0.209
28	Coumarin	20.9	ND	56	Acacetin	40.7	0.019

RT: Retention time; ND: Not detected; NA: Not applicable; IS: Internal standard

### Effects of SP extract on live body weight and fasting blood glucose level

The live body weight (LBW) of the control group and the DM + SP-100 mg/kg extract group at the end of the experiment was significantly higher than the measurements on the first day (Table 2) (P < 0.0001). The fasting blood glucose (FBG) level of the DM group was statistically higher on the last day of the experiment than on the first day (P < 0.05). On the other hand, the SPE treatment (400 mg/kg) caused a significant decrease in the FBG level at the last measurement compared with the first (Table 2) (P < 0.05).

**Table 2:** Changes in live body weight and fasting blood glucose level measurements between the beginning and the final

Parameters	Control	DM	DM + SP-100	DM + SP-200	DM + SP-400	DM + Gly-3
**Live body weight (g)**						
*Beginning*	208.14 ± 5.64	216.57 ± 4.27	182.14 ± 5.02	187.71 ± 5.63	195.86 ± 3.60	181.14 ± 5.54
*Final*	266.14 ± 7.70^****^	243.71 ± 12.37	212.14 ± 13.73^*^	201.43 ± 15.47	200.14 ± 13.51	183.71 ± 8.53
**Fasting blood glucose level (mg/dL)**						
*Beginning*	95.14 ± 3.09	445.86 ± 28.33	416.71 ± 15.71	419.43 ± 22.60	371.57 ± 24.09	309.71 ± 24.11
*Final*	102.57 ± 3.89	517.29 ± 26.88^*^	445.00 ± 44.03	435.00 ± 39.21	281.71 ± 15.27^*^	316.57 ± 31.29

Data are presented as mean ± standard error of mean (SEM). *p < 0.05, ****p < 0.0001: Significant compared to the beginning; DM: Diabetes mellitus; SP: *Scutellaria pinnatifida;* Gly: Glibenclamide

### Effects of SP extract on serum biochemical parameters

**Fig. 2:**
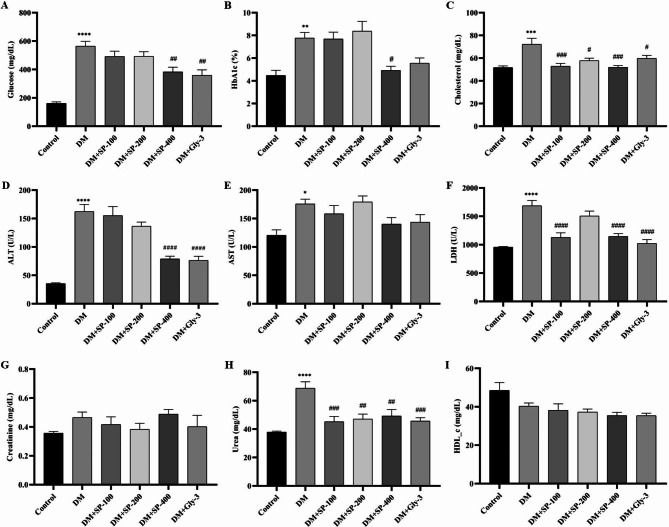
The effect of SP extract on HbA1c and serum biochemical parameters. **(A)** Glucose levels. **(B)** HbA1c levels. **(C)** Cholesterol levels. **(D)** Alanine aminotransferase (ALT) levels. **(E)** Aspartate aminotransferase (AST) levels. **(F)** Lactate dehydrogenase (LDH) levels. **(G)** Creatinine levels. **(H)** Urea levels. **(I)** High-density lipoprotein cholesterol (HDL_c) levels. Data are presented as mean ± standard error of mean (SEM). Control vs. DM: ^*^P < 0.05, ^**^P < 0.01, ^***^P < 0.001 and ^****^P < 0.0001; DM vs. all treatment groups: ^#^*P* < 0.05, ^##^*P* < 0.01, ^###^*P* < 0.001 and ^####^*P* < 0.0001

STZ induction caused a significant increase in serum glucose level according to control (P < 0.0001). However, the treatments with 400 mg/kg SPE and Gly significantly decreased glucose levels according to DM (Fig. 2) (P < 0.01). HbA1c % of DM was measured significantly higher than control (P < 0.01). In contrast, SPE-400 treatment resulted in a significant decrease in HbA1c % according to DM (P < 0.05). STZ induction caused a significant increase in ALT level in serum according to control (P < 0.0001). However, the treatments with 400 mg/kg SPE and Gly resulted in a statistical decrease in ALT level according to DM (P < 0.0001) (Fig. 2). The AST level of DM was significantly higher than in the control group (P < 0.05). Serum cholesterol and urea levels in the DM group were higher than those in the control and treatment groups (Fig. 2). On the other hand, LDH levels were significantly lower in the DM + SP-100, DM + SP-400 and DM + Gly groups than in the DM group (P < 0.0001) (Fig. 2). The HDL_c level in the DM group was found to be lower than the control group (Fig. 2).

### Effects of SP extract on MDA, GSH levels and antioxidant enzyme activities

The MDA content in liver, kidney and pancreas tissues of the DM group was significantly higher than in control animals. Significant reductions in MDA content in the treatment groups were found compared to the DM group, especially in the SPE-400 (P < 0.05) and Gly (P < 0.01) treated groups (Table 3). GSH levels in the liver and pancreas of the DM group were importantly lower than those of the control group. On the other hand, treatment with 400 mg/kg SPE resulted in a statistical increase in GSH levels compared to the DM group (Table 3) (P < 0.05). Treatment with SPE-400 resulted in a significant increase in GST enzyme activity in pancreatic tissue compared with the untreated diabetic group (P < 0.01). In addition, GR activity in liver tissue of the DM + SPE-400 group was significantly higher than that of the DM group (P < 0.01). The significant increase of the CAT activity in pancreatic tissues of treated groups except SPE-100 was found in the DM group (P < 0.0001). Moreover, the treatments with SPE-200 and Gly promoted the SOD activity in pancreatic tissues in the DM group (P < 0.05). Furthermore, Gly resulted in a significant increase in GPx activity in the liver and pancreas according to DM group (P < 0.05) (Table 3).

**Table 3:** The effect of SP extract on MDA, GSH contents and antioxidant enzyme activities in various tissues

Tissues	Parameters	Control	DM	DM + SP-100	DM + SP-200	DM + SP-400	DM + Gly-3
**Liver**	MDA (nmol/g prt)	0.61 ± 0.02	0.76 ± 0.02^**^	0.70 ± 0.04	0.60 ± 0.02^##^	0.63 ± 0.02^#^	0.62 ± 0.01^##^
	GSH (GSH/mg prt)	92.56 ± 1.46	76.79 ± 3.06^****^	84.93 ± 1.82	80.65 ± 1.36	85.23 ± 2.10^#^	85.07 ± 1.22^#^
	GST (nmol/g prt)	155.83 ± 2.17	144.14 ± 2.43	148 ± 6.44	138.79 ± 3.56	162.24 ± 5.32	149.61 ± 3.72
	GR (U/g prt)	2.90 ± 0.06	2.61 ± 0.11	2.83 ± 0.07	2.83 ± 0.12	3.13 ± 0.10^##^	2.87 ± 0.05
	CAT (nmol/g prt)	24.71 ± 1.13	20.93 ± 0.61^*^	22.42 ± 0.93	19.39 ± 0.98	20.65 ± 0.60	21.01 ± 0.60
	SOD (pg/mg prt)	266.19 ± 19.95	223.40 ± 18.40	233.83 ± 11.82	295.97 ± 57.52	246.97 ± 19.03	226.47 ± 9.25
	GPx (IU/mg prt)	34.24 ± 4.04	29.3 ± 0.46	36.02 ± 2.77	32.4 ± 3.90	38.13 ± 2.02	44.08 ± 3.74^#^
**Kidney**	MDA (nmol/g prt)	1.21 ± 0.06	1.63 ± 0.04^****^	1.32 ± 0.07^###^	1.39 ± 0.03^#^	1.27 ± 0.04^###^	1.27 ± 0.04^####^
	GSH (GSH/mg prt)	119.81 ± 6.45	108.04 ± 3.53	115.47 ± 3.04	119.46 ± 1.32	119.48 ± 3.01	119.52 ± 3.67
	GST (nmol/g prt)	19.01 ± 0.33	17.55 ± 0.88	18.16 ± 1.17	20.40 ± 1.12	20.15 ± 0.29	17.20 ± 0.93
	GR (U/g prt)	6.38 ± 0. 18	5.73 ± 0.17	6.23 ± 0.26	6.11 ± 0.21	5.77 ± 0.19	6.07 ± 0.23
	CAT (nmol/g prt)	12.86 ± 0.52	10.73 ± 0.51	10.79 ± 0.49	11.80 ± 0.39	11.25 ± 0.51	11.67 ± 0.51
	SOD (pg/mg prt)	678.68 ± 60.81	429.74 ± 24.48^**^	455.23 ± 33.41	523.70 ± 33.56	473.36 ± 26.67	459.07 ± 45.30
	GPx (IU/mg prt)	105.83 ± 15.09	61.66 ± 4.04^**^	63.02 ± 4.28	75.08 ± 5.84	68.20 ± 4.31	82.25 ± 7.20
**Pancreas**	MDA (nmol/g prt)	3.09 ± 0.14	4.45 ± 0.19^**^	4.30 ± 0.15	4.10 ± 0.31	3.21 ± 0.40^#^	3.40 ± 0.24
	GSH (GSH/mg prt)	173.13 ± 11.26	89.98 ± 9.22^****^	105.50 ± 7.23	129.66 ± 12.46	149.49 ± 13.76^##^	131.23 ± 8.15
	GST (nmol/g prt)	25.69 ± 2.13	15.61 ± 1.22^*^	13.43 ± 0.93	23.32 ± 2.92	28.32 ± 2.55^##^	21.25 ± 1.31
	GR (U/g prt)	7.08 ± 0.42	5.33 ± 0.46	7.08 ± 0.50	5.18 ± 0.46	6.54 ± 0.29	5.94 ± 0.38
	CAT (nmol/g prt)	4.27 ± 0.14	1.71 ± 0.12^****^	2.22 ± 0.13	3.82 ± 0.18^####^	3.84 ± 0.31^####^	3.66 ± 0.25^####^
	SOD (pg/mg prt)	1509.41 ± 118.91	1485.23 ± 162.85	2184.34 ± 276.28	2501.17 ± 270.30^#^	1712.02 ± 187.39	2575.47 ± 263.70^#^
	GPx (IU/mg prt)	185.71 ± 16.40	180.51 ± 8.49	239.86 ± 34.13	282.10 ± 26.28	230.63 ± 27.36	324.05 ± 37.99^#^

Data are presented as mean ± standard error of mean (SEM). Control vs. DM: ^*^P < 0.05, ^**^P < 0.01, ^***^P < 0.001 and ^****^P < 0.0001; DM vs. all treatment groups: ^#^*P* < 0.05, ^##^*P* < 0.01, ^###^*P* < 0.001 and ^####^*P* < 0.0001. MDA: Malondialdehyde; GSH: Reduced glutathione; GST: Glutathione-S-transferase; GR: Glutathione reductase; CAT: Catalase; SOD: Superoxide dismutase; GPx: Glutathione peroxidase; DM: Diabetes mellitus; SP: *Scutellaria pinnatifida;* Gly: Glibenclamide

### Histopathological examinations on liver, kidney and pancreas

**Fig. 3:**
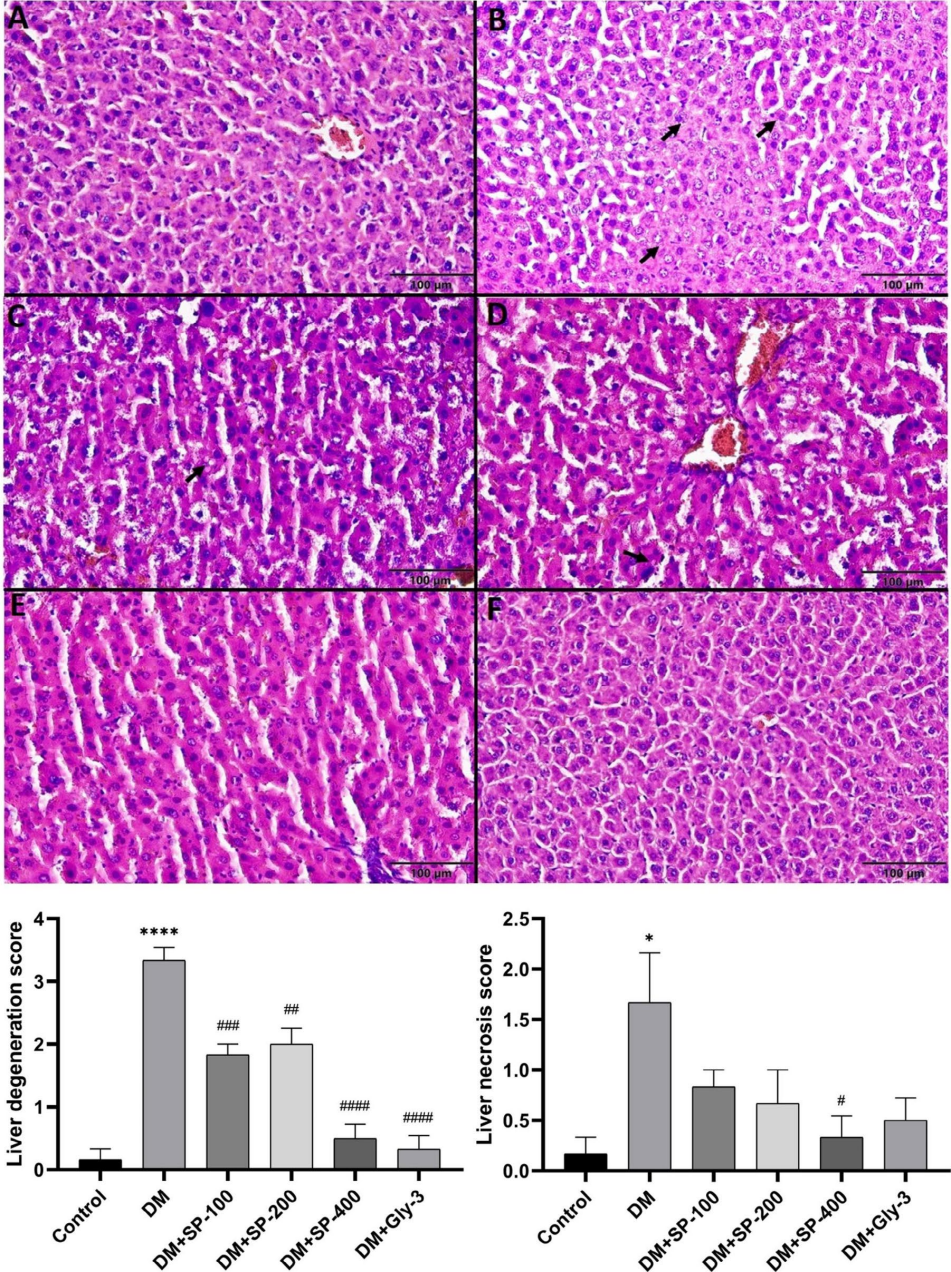
Microscopic image of the liver tissue. The liver tissue in the control **(A)** group was a normal histological architecture, the liver in the DM **(B)** group had severe degeneration (arrow) and moderate necrosis (arrowhead), DM + SP-100 **(C)** and DM + SP-200 **(D)** groups had mild degeneration (arrow), DM + Gly **(F)** and DM + SP-400 **(E)** groups had normal histological architecture. H&E. Data are presented as mean ± standard error of mean (SEM). Control vs. DM: ^*^P < 0.05, ^**^P < 0.01, ^***^P < 0.001 and ^****^P < 0.0001; DM vs. all treatment groups: ^#^*P* < 0.05, ^##^*P* < 0.01, ^###^*P* < 0.001 and ^####^*P* < 0.0001

**Fig. 4:**
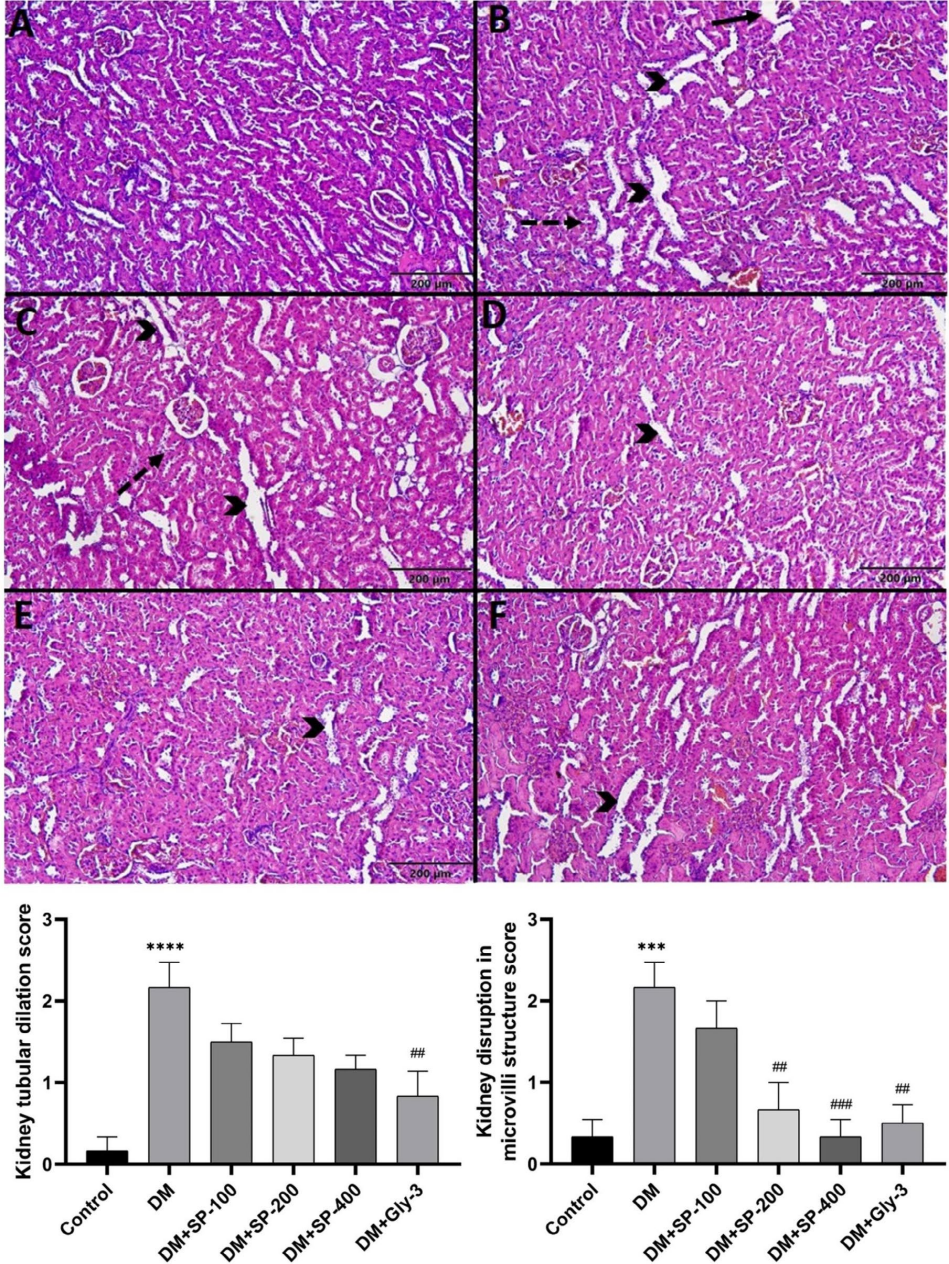
Microscopic image of kidney tissue. The kidney tissue of the control group had normal histological architecture **(A)**. Moderate tubular dilatation (arrowhead) was observed in the DM **(B)** and DM + SP-100 **(C)** groups, while mild tubular dilatation was observed in the DM + Gly **(F)**, DM + SP-200 **(D)** and DM + SP-400 **(E)**. The filtration space of the DM and DM + SP-100 groups enlarged. The integrity of microvilli in the tubular epithelium of the kidneys (dashed arrow) in the DM and DM + SP-100 groups was moderately impaired. H&E 400x. Data are presented as mean ± standard error of mean (SEM). Control vs. DM: ^*^P < 0.05, ^**^P < 0.01, ^***^P < 0.001 and ^****^P < 0.0001; DM vs. all treatment groups: ^#^*P* < 0.05, ^##^*P* < 0.01, ^###^*P* < 0.001 and ^####^*P* < 0.0001

**Fig. 5:**
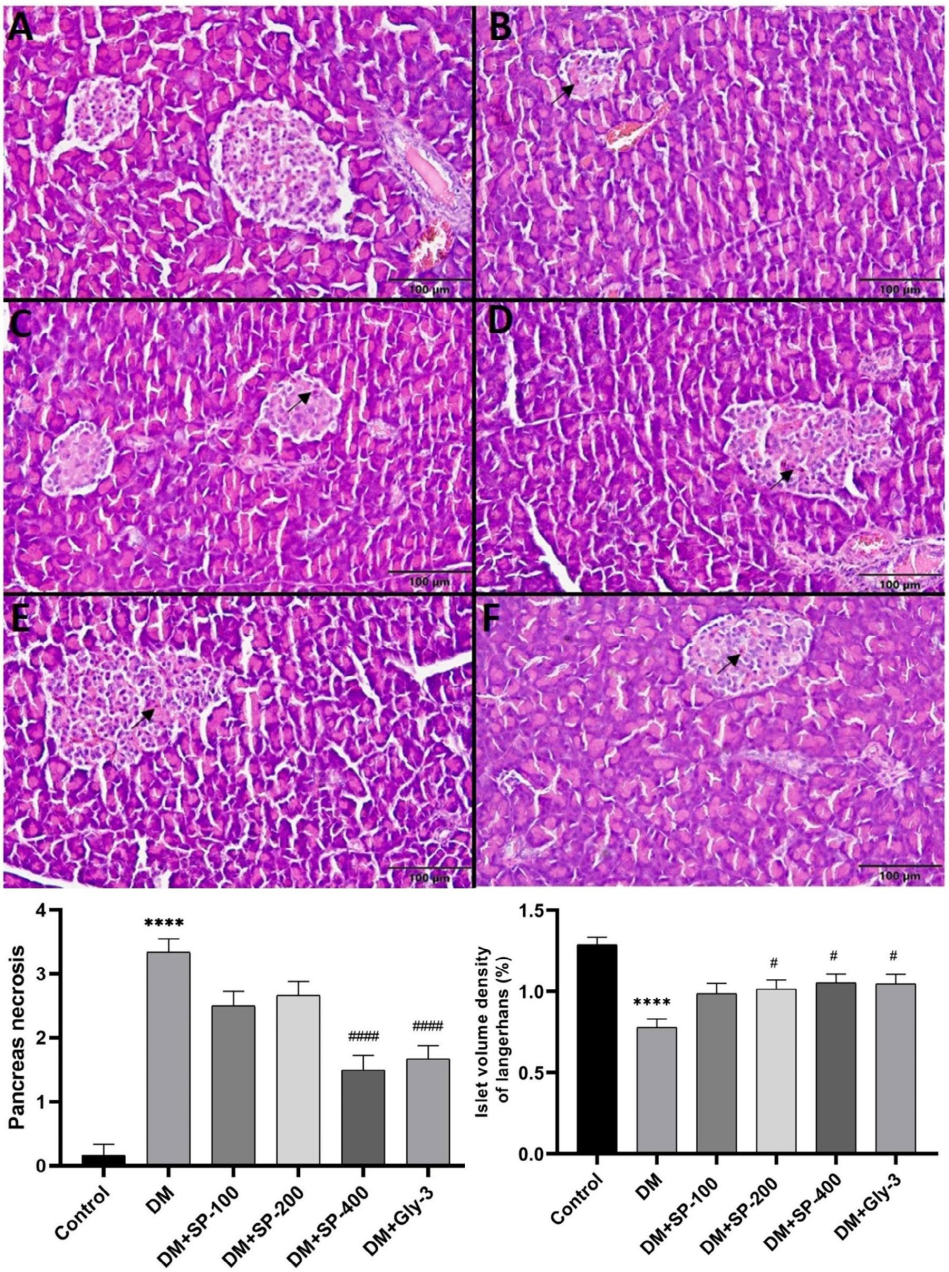
Microscopic image of pancreatic tissue. Control group had normal histological architecture **(A)**. Severe necrotic cells were found in the DM **(B)**, DM + SP-100 **(C)**, and DM + SP-200 **(D)**, moderate necrotic cells were found in the DM + SP-400 **(E)** and DM + Gly **(F)** groups. H&E 400x. Data are presented as mean ± standard error of mean (SEM). Control vs. DM: ^*^P < 0.05, ^**^P < 0.01, ^***^P < 0.001 and ^****^P < 0.0001; DM vs. all treatment groups: ^#^*P* < 0.05, ^##^*P* < 0.01, ^###^*P* < 0.001 and ^####^*P* < 0.0001

Liver, kidney and pancreas were examined and scored histopathologically (Figs. 3, 4 and 5). The liver tissue in the control group had normal histological architecture (Fig. 3A). A significant increase in liver degeneration was observed in the DM group compared with the control group (P < 0.0001) (Fig. 3). However, all of the treatment groups showed a significant decrease in liver degeneration, compared with the diabetic control (Fig. 3). In addition, a significant improvement in liver degeneration was observed in the DM + SP-400 and DM + Gly groups according to DM group (P < 0.0001) (Fig. 3). A significant increase in liver necrosis in the DM group compared with the control group (P < 0.05). Treatment with 400 mg/kg SP resulted in an improvement in liver necrosis with the diabetic control group (P < 0.05) (Fig. 3).

The kidney tissue in the control group had normal histologic architecture (Fig. 4A). A significant increase in the kidney tubular dilation was observed in DM group compared with the control group (P < 0.0001) (Fig. 4). In addition, a significant improvement in kidney tubular dilation was observed in the DM + Gly group compared with the DM group (P < 0.01) (Fig. 4). Kidney disruption in microvilli in DM (P < 0.001) was higher than in normal controls. On the other hand, a significant decrease in kidney disruption in microvilli was observed in DM + SP-200 (P < 0.01), DM + SP-400 (P < 0.001) and DM + Gly (P < 0.01), compared with the DM group (Fig. 4).

The pancreas of rats in the control group had normal histologic architecture (Fig. 5A). A significant increase in the pancreatic necrosis in the DM group was observed compared with the control group (P < 0.0001) (Fig. 5). 400 mg/kg SP and 3 mg/kg Gly treatments resulted in an improvement in pancreatic necrosis compared with the diabetic control group (P < 0.0001). A significant decrease in the islet volume density of Langerhans in DM group was observed compared with the control group (P < 0.0001) (Fig. 5). On the other hand, a significant increase in the islet volume density of Langerhans was observed in DM + SP-200, DM + SP-400 and DM + Gly groups compared with the diabetic control group (P < 0.05) (Fig. 5).

## Discussion

In this study, the bioactive phenolic compounds were detected and quantified by LC-MS/MS analysis in SPE. In addition, the antioxidant and antidiabetic properties of SPE were investigated. Therefore, we investigated the curative effect of SPE on oxidative stress and histopathological changes in liver, kidney and pancreas, as well as on biochemical parameters in the serum of STZ-induced diabetic rats.

*Scutellaria* species contain a high concentration of phenolic and flavonoid compounds. In the ethyl acetate extract of *S. orientalis* subsp pinnatifida, an extract yield of 21%, a total phenolic content of 160.00 ± 1.91 and a total flavonoid content of 74.56 ± 0.53 were detected [48]. The extract yield, total phenolic and total flavonoid contents in this study are in agreement with previous studies. Also, in our study, we found that the ethanol extract of *S.pinnatifida* contains mainly apigenin as well as luteolin, quinic acid, cosmosiin and epigallocatechin (Fig. 1; Table 1). The presence of apigenin and luteolin in *Scutellaria* species has been reported [25, 29]. Our findings obtained from LC-MS/MS analysis are in agreement with previous studies.

The loss of the LBW and high FBG levels are the most prominent symptoms observed in diabetes patients. The current study demonstrated that administration of the highest dose of SPE for 21 days showed antihyperglycemic effect and balanced LBW in STZ-induced diabetic rats (Table 2). Extracts and monomeric compounds isolated from *Scutellaria* species have antidiabetic properties [49]. The change of LBW in diabetic rats may show differences depending on the duration of treatment and dose [50]. Moreover, our initial and final measurement results for the parameter LBW were consistent with the diabetes model performed on *Scutellaria baicalensis* ob/ob mice [51]. The bioactive components of the extract could contribute to the significant reduction of FBG and stabilization of LBW in SPE-treated diabetic rats by stimulating insulin secretion from the pancreatic β-cells.

DM is characterized by high blood glucose and HbA1c levels. According to our results, the highest SPE concentration resulted in a decrease in serum glucose and HbA1c levels (Fig. 2). Similar to our results, the plant extracts of *Cyanus depressus* [38] and *Achillea arabica* [52] caused a decrease in blood glucose and HbA1c levels in the STZ-induced diabetic rat model. In addition, DM not only increases blood glucose levels, but also has negative effects on biomarkers in the liver and kidney and parameters of the lipid profile. HDL_c levels decrease in diabetic patients compared to non-diabetics [53]. Similarly, STZ induction in our study resulted in a decrease in HDL_c in diabetic groups. However, it was insignificant in DM group according to the control group (Fig. 2). STZ-induced OS leads to degradation of lipids, proteins and nucleic acids in both the liver and kidneys as a result of increased ROS. ROS destroys the unsaturated fatty acids of the cell membrane, causing the cell’s enzymes to leak into the bloodstream. Measurement of this leakage provides useful data on the liver and kidney damage. According to our results, SPE-400 treatment improved ALT, LDH and urea levels in STZ-induced diabetic rats, similar to the results of the glibenclamide treated group. In addition, SPE and glibenclamide contributed to the reduction of cholesterol levels in the DM groups (Fig. 2). These beneficial effects may be due to the stimulatory effects of apigenin, luteolin, quinic acid, cosmosiin and epigallocatechin in SPE. These phytochemical compounds have antidiabetic, hepatoprotective and nephroprotective effects in STZ-induced diabetic rats [38, 54, 55].

An imbalance in cellular reduction oxidation leads to OS and subsequently to the onset and development of diabetes and related complications by affecting certain signaling pathways involved in β-cell dysfunction and insulin resistance [56]. ROS affects specific proteins and lipids involved in the progression of diabetes, triggering protein oxidation and lipid peroxidation. Elevated OS causes free radicals to attack proteins, carbohydrates, lipids, and nucleic acids, and leads to a decrease in enzyme activities of antioxidant defense systems, and an increase in lipid peroxidation and histopathological distortions [38]. The MDA content in the liver, kidney and pancreas tissues of the DM group was significantly higher than that of the control group. SPE treatment prevented lipid peroxidation by decreasing the MDA content in the liver, kidney and pancreas of STZ-induced rats, especially in the SPE-400 and Gly treated groups (Table 3). In addition, we found that SPE-400 treatment positively affected the levels of GSH (in liver and pancreas), a nonenzymatic antioxidant, and GST enzyme activity (in pancreas). Moreover, the SPE treatment groups were found to have positive effects on CAT, GR, SOD and GPx activities, although they showed variations depending on the dose and tissue type (Table 3). Furthermore, the higher doses of SPE and Gly treatment produced histopathological improvements in agreement with our biochemical parameters (Figs. 3, 4 and 5). Percolation and pressurized liquid extraction of SP root extract, which is a rich natural source of antioxidants and flavonoids, has a reducing antioxidant effect on iron (FRAP) and 1,1-diphenyl-2-picrylhydrazyl (DPPH) radical scavenging activity under in vitro conditions [18]. In another study, ethanolic root extract of *Scutellaria baicalensis* showed ameliorative effect on STZ-induced liver, kidney and pancreas injury in rats based on liver biomarkers and lipid peroxidation, antioxidant enzyme activities and histopathological findings [57]. Hyperglycemia mainly causes damage to the hepatic, renal and pancreatic systems. In STZ-induced diabetes, hepatocytes typically show focal vacuolar degeneration, hypertrophy, sinusoidal dilation, focal necrosis and apoptosis [58]. Our histopathological findings of the liver, kidney and pancreas (Figs. 3, 4 and 5) were in agreement with the results in the literature [38]. The density of islets of Langerhans was also significantly greater in all treatment groups than in the DM group (Fig. 5). STZ caused a decrease in the volume of islets of Langerhans and an increase in necrosis in pancreatic β-cells in diabetic rats [59]. *Scutellaria baicalensis*, which belongs to the same genus as our plant, caused an increase in the volume of pancreatic islets of Langerhans in STZ-induced diabetic rats [57]. In light of the antidiabetic, antioxidant and histopathological results of this study, SPE had ameliorative effects in STZ-induced diabetic rats due to the high content of phytochemicals in the extract. Apigenin, rutin, cosmosiin, luteolin, cynaroside and epigallocatechin are found to be major compounds in SP plant. In this regard, their antioxidant, antidiabetic, antihyperlipidemic, histopathological reducing cell degeneration and lipid peroxidation inhibitory activities have been reported [60–62].

## Conclusion

The major chemical compounds found in SPE, including apigenin, luteolin, quinic acid, cosmosiin and epigallocatechin were identified and quantified by LC-MS/MS analysis. SPE treatment decreased glucose and HbA1c levels in STZ-induced diabetic rats. In addition, SPE treatment reduced lipid peroxidation and improved indicators of liver and kidney damage. Moreover, SPE showed antioxidant activity in the liver, kidney and pancreas tissues of diabetic rats. Furthermore, histopathological findings in the liver, kidney and pancreas confirmed our results. In conclusion, the above-ground ethanolic extract of *Scutellaria pinnatifida* has antidiabetic and antioxidant potential due to its high content of phytochemicals.

## Data Availability

The datasets generated during and/or analyzed during the current study are available from the corresponding author on reasonable request.

## Abbreviations

ALT: Alanine aminotransferase
AST: Aspartate aminotransferase
CAT: Catalase
DM: Diabetes mellitus
FBG: Fasting blood glucose
Gly: Glibenclamide
GPx: Glutathione peroxidase
GR: Glutathione reductase
GSH: Reduced glutathione
GST: Glutathione *S*-transferase
HbA1c: Glycosylated hemoglobin
HDL_c: High-density lipoprotein cholesterol
LBW: Live body weight
LC–MS/MS: Liquid chromatography tandem mass spectrometry
LDH: Lactate dehydrogenase
MDA: Malondialdehyde
OS: Oxidative stress
RNS: Reactive nitrogen species
ROS: Reactive oxygen species
SOD: Superoxide dismutase
SP: *Scutellaria pinnatifida*
SPE: *Scutellaria pinnatifida* extract
STZ: Streptozotocin
TC: Total cholesterol
